# A qualitative study of naturopathy in rural practice: A focus upon naturopaths' experiences and perceptions of rural patients and demands for their services

**DOI:** 10.1186/1472-6963-10-185

**Published:** 2010-06-28

**Authors:** Jon L Wardle, Jon Adams, Chi-Wai Lui

**Affiliations:** 1 School of Population Health, University of Queensland, Public Health Building, Herston Rd, Herston, 4006, Australia; 2 The Network of Researchers in the Public Health of Complementary and Alternative Medicine (NORPHCAM), School of Population Health, University of Queensland, Public Health Building, Herston Rd, Herston, 4006, Australia

## Abstract

**Background:**

Complementary and alternative medicine (CAM) use - of which naturopathy constitutes a significant proportion - accounts for approximately half of all health consultations and half of out-of-pocket expenditure in Australia. Data also suggest CAM use is highest amongst rural Australians. Unfortunately little is known about the grass-roots reality of naturopathy or other CAM use in rural regions.

**Methods:**

Semi-structured interviews were conducted with 20 naturopaths practising in the Darling Downs region of South-East Queensland to assess their perceptions and experiences of rural patients and demand for their services.

**Results:**

Naturopaths described strong demand in rural areas for their services and perceived much of this demand as attributable to cultural traits in rural communities that served as pull factors for their naturopathic services. Such perceived traits included a cultural affinity for holistic approaches to health and disease and the preventive philosophy of naturopathy and an appreciation of the core tenet of naturopathic practice to develop closer therapeutic relationships. However, cost and a rural culture of self-reliance were seen as major barriers to naturopathic practice in rural areas.

**Conclusions:**

Demand for naturopathic services in rural areas may have strong underlying cultural and social drivers. Given the apparent affinity for and increasingly large role played by CAM services, including naturopathic medicine, in rural areas it is imperative that naturopathic medicine and the CAM sector more broadly become a core focus of rural health research.

## Background

Complementary and alternative medicine (CAM) practitioner consultations constitute approximately half of total health consults and more than half of out-of-pocket healthcare costs in Australia [1]. Data from Western Australia suggest that more than half of all health consults in the rural South-West region of the state are with CAM practitioners [2]. High CAM use has also been identified in other rural areas throughout Australia [3-7]. Alongside similar prevalence of CAM use in rural regions identified overseas [8-14] these findings have prompted some researchers to suggest geographical location, in particular the urban/rural divide, as one important factor in predicting CAM use [14-17]. This has led to further calls attention upon geographical location within future CAM consumption research [18-20].

CAM use has emerged as a significant public health issue [21] and there is a need to examine and understand its role in rural health care delivery in order to aid effective, coordinated care and to inform evidence-based health policy [18]. This need is made ever more urgent and significant when considered alongside a number of challenges facing contemporary rural health care delivery and provision in Australia and elsewhere: the uneven distribution and relative shortage of medical care providers (particularly general practitioners but also allied health professionals) in rural areas [22,23]; and the closely associated problems of recruitment and retention of primary care practitioners to the rural workforce [24-26]. The largely unexamined (at least in terms of official health policy and research) network of CAM and CAM practitioners, such as naturopaths, may currently fill service gaps and provide essential support to rural patients [27,28].

Unfortunately, we know very little about the grass-roots reality of CAM use and provision in rural areas. Most of the available studies on this topic focus on the prevalence of CAM use and provide only a descriptive account of categories of CAM consumption, especially among older rural adults [13,29-35] or ethnic minority groups [36-38]. Most importantly, the bulk of work has targeted patients/users as a subject of investigation and there is a lack of exploration of CAM practitioner experience in rural health and welfare practice [18,39].

In response, this paper reports findings from an exploratory qualitative study examining the perceptions and experiences of rural naturopaths with regard to their patients and the demand for their services. The study provides a first step towards better understanding of the role of naturopathy 'on the ground' in rural health care.

## Methods

Qualitative semi-structured interviews were chosen for data collection due to the lack of previous research on this topic and the related need for an exploratory approach to investigate CAM research in the rural setting [40]. Ethical approval for the study was obtained from the School of Population Health Research Ethics Committee, University of Queensland in accordance with the guidelines set by the National Health and Medical Research Council.

Study participants were selected from naturopaths currently practicing in the Darling Downs region of South East Queensland who registered with either one of Australia's 'big four' accrediting professional associations for naturopaths (i.e., the *Australian Natural Therapist's Association*; the *Australian Naturopathic Practitioners Association*; the *Australian Traditional Medicine Society; *and the *National Herbalist's Association of Australia*). The Darling Downs region consists of the Toowoomba, Goondiwindi, Southern Downs and Western Downs regional councils and is a predominantly agricultural area which comprises of a population of 227,074 in an area of 77,424 km^2^[41]. Although a great level of heterogeneity exists between specific areas, the region as a whole has greater rates of socio-economic disadvantage, lower cultural and ethnic diversity and reduced access to services than are commonly observed in rural areas throughout Australia [41,42]. Participants were initially contacted using the online practitioner databases administered by the above professional associations [43-46]. The focused study area allowed all practitioners in the area to be contacted and all practitioners that expressed interest in participating to be interviewed. A table of the sample compared to the Australian naturopathic practitioner workforce is available in Table 1.

**Table 1:** Sample profile compared to all Australian naturopaths

	Australian Average*	Sample population
Average Age	44.1	39.2

% Female	76%	55%

No of Years in Practice	6.7 years	8.2 years

Average Training Length	3.1 years	3.5 years

Weekly Hours in Clinic	24.8 hrs	32.1 hrs

*Average data for Australian naturopathic workforce taken from Lin V, Bensoussan A, Myers SP, McCabe P, Cohen M, Hill S, Howse G: The practice and regulatory requirements of naturopathy and western herbal medicine. Melbourne: Victorian Department of Human Services, 2005 [65]

All interviews were completed at a place and time convenient for the participant. The participants were provided with information of the research and asked to sign a consent form prior to interview. All interviews were audiotaped and lasted between 40 and 100 minutes (60 minutes average). A list of questions (Additional File 1: Table S1) was prepared to guide the interview but the participants were allowed to direct and shape the discussion in line with their focus and concerns. Keywords, phrases and arguments used by the naturopath were noted and their meanings clarified as the interview proceeded.

In line with other qualitative studies, interview fieldwork was terminated only after thematic saturation was achieved [47] - with new cases producing no new significant themes. As all naturopaths practising in the study area had been offered the opportunity to be interviews this resulted in 20 completed naturopath interviews.

All tapes were transcribed to computer files shortly following interview. The process of transcription was concurrent to the processes of data collection and preliminary analysis with codes and analytical themes developed in a cumulative manner. These codes and themes were then fed back into the coding process. The method of researcher triangulation was also employed with each member of the research team providing independent analysis of selections of transcription and compared the results afterward.

## Results

Data analysis identified a number of core themes from the naturopaths' accounts of their rural practice and patients. These themes have been grouped for heuristic reasons under two broad headings and reported here. Direct quotations are provided to illustrate these themes and to ensure rigor in the reporting of data. To avoid identifying specific individuals, the participants have been allocated pseudonyms.

### 1. Explaining the demand for naturopathy in rural health: affinity with rural patients and populations

The participants explained how their practice constituted a popular treatment option attracting good numbers of local and regional patients. As the quotes below illustrate, the naturopaths often made reference to what they perceived as the 'open-minded' and/or 'independent' outlook or approach of rural patients as a factor for demand for their services in the bush:

'I think country people are generally more open-minded and less cynical about things than city people... so I think they're more willing to give things a go.' (DR)

'I don't think they're exposed to as much of the negativity as city people... you know they're not bombarded with media or advertising so they have to make up their own minds.' (MC)

These perceptions were further explained by some naturopaths in terms of the stoic approach of rural patients not just to health but also to life generally. As the quotes below illustrate, this stoic approach is often associated with a preference for preventive measures and self-care - a preference that naturopaths' claim fits comfortably with their perspective and practice:

'[Country people] are very stoic... they're brought up with this idea that you don't seek help until the last minute... and I think they enjoy the fact that you teach them to look after themselves rather than telling [them] what to do.' (JD)

'I really think that that preventative role of naturopathy can really help country people... they work themselves into the ground so much until... they die ... and they aren't often able to get away from their farms to see you. So if you can teach them to look after themselves... they really appreciate that aspect.' (SJ)

As this last quote suggests, the constraints and logistics of rural work and life may also be instrumental in establishing a convenient affinity between the preventive and empowering tenets of naturopathic care and the needs and abilities of rural patients with regard to accessing health services.

Some naturopaths pointed to the extensive time commitment and support provided by naturopathic consultations as another 'pull factor' for rural patients in seeking their services. As the following quotes illuminate, these claims referred to logistics and the commitments associated with accessing health care in rural locations:

'Some of these people are driving seven or eight hours to see a healthcare professional... [if the consultation only lasts for] 15 minutes and [costs] 200 bucks they get very disappointed... I think that's why they like coming to see someone like us... we try to make the trip worthwhile.' (DR)

'[Country people] have a larger sense of commitment... I've found that if I... include their family members and include their story... that's half their healing.... They don't get heard in the medical establishment because it's 5 minutes, 15 minutes if they're lucky... I've found that country people want to talk first.' (PE)

Likewise, the quote below pointed to the affinity with closer personal relationships (including those between patient and practitioner) among rural populations as cementing the demand for their naturopathic services:

'They're more inclined to see me because I listen... and because I remember the story the next time they come... or if I meet them down the street they will tell me... and I have to remember those little pieces... it's very different [from patient-practitioner relationships in urban communities].' (LS)

Others suggested that another key factor driving rural patients to seek complementary therapists was the fact that complementary therapists, more so than conventional health practitioners, were often originally from the areas they practised in, with deep cultural roots in the community. Practitioners not originally from the area often mentioned that they were only accepted after they had become 'locals'. This connection to and understanding of the culture of the community may help deeper therapeutic relationships with their patients, as illustrated in this quote:

'I definitely think that the fact that I'm originally from here made a huge difference... patients automatically feel more comfortable because they know you're aware of issues that arise from living in the area... I think if I was someone coming up from [the city] they wouldn't have been anywhere near as open to start with' (WX)

This connection to the community was perceived to be more important than the type of practitioner they were. The community perception of who is a 'foreign doctor' could apply equally to a medical graduate from Brisbane, Sydney or Melbourne as it could to an international medical graduate. However, international medical graduates (who make a significant proportion of the Australian rural medical workforce) may present issues of their own in relation to local complementary medicine use that disconnected them from their patients, as the following quote illustrates:

'In rural areas a lot of the doctors are foreign so maybe they don't understand, or are suspicious of complementary medicines... not suspicious as in they don't believe in it but maybe they come from places where they've not had any contact with the complementary therapies or naturopaths the locals use' (EM)

Not only do close therapeutic relationships serve as significant pull factors for naturopathic consultations, in some patients they also serve to differentiate the naturopaths from conventional medical practitioners, as this quote from a participant illustrates:

'It's always "she doesn't listen" or "he doesn't listen" or "they've always got their face on the computer", or "how do they know me if they don't talk to me"... There's always a bit of a whinge first [about how the doctor treated them last time]... people come to see me because I'm not a doctor'. (PE)

Meanwhile, other naturopaths appeal to a notion of historical affinity to explain the high use of naturopathy in rural populations. As one participant suggested:

'Western [rural] people are far more open... natural therapies have always been a part of their lives... some of the founding fathers of naturopathy started out in places like this... that culture is still alive here... it never died out.' (SJ)

And another explained:

'I think people in the country still have access to all those old grandma cures... I mean they haven't lost that part of their culture like people in the city have.' (DH)

Linked to this notion of rural communities as characterised by closer personal relations, some naturopaths highlighted how both rural patients and naturopaths share a broader conception of health and illness, one that places emphasis on family, community and social ties and acknowledges the relationship between health and wider environment. This conception of health is characterised by these practitioners as holistic and supporting a version of holistic practice:

'[Rural people] understand the importance of social contact and things like that... in [regional city] they're too busy... country people... I would see that they embrace holism more... but the theory of it may not be talked about.' (WX)

'I think that rural people are more accepting of holism... I don't think they'd know what the word means... but they're aware of the fact that... your social situation or the fact that you had a bad crop can be bad for your health.... Everyone knows each other's stories... so when you hear bad news about someone... and then they have health problems as well... well... people put two and two together.' (KJ)

The naturopaths' explanations of this pragmatic attitude towards the complex interactions and inter-connections affecting health seems to stem more from lived experiences and attitudes in rural areas than from new age or esoteric philosophies often attributed as pull factors for CAM in the broader community:

'The people here are much closer to nature... the natural cycle... they believe in the power of it... they see things get born... get sick... die... they're just more earthier people' (DL)

However, not all explanations of naturopathic practice in rural communities were couched in entirely positive terms and as the next section of analysis outlines, there were a number of perceived challenges associated with providing naturopathic services to the wider rural community.

### 2. Barriers and challenges to naturopathy use in rural health

Despite their overall presentation of naturopathy as well suited to rural patients and populations, the naturopaths also explained how such suitability does not necessarily translate into straightforward patient recruitment.

Several practitioners outlined how the resourcefulness and independence of rural people may produce challenges in providing timely treatment - a difficulty acknowledged as facing both conventional and complementary practitioners - but were particularly a problem for naturopaths working in the competitive private sector where treatment costs are entirely out-of-pocket and borne solely by the patient. As one naturopath explained:

'In [the city] I found people were much happier paying for the service whereas in [regional country town] people are much happier getting as much as they can for less... they'll ask someone at the health food store but it's hard to get them to come and see me.' (KJ)

Similarly, another naturopath described how rural people are 'proud' and how their self-reliance can be a challenge to providing care. This can be seen as the flip side to the positive affinity between a patient's self-reliance and a naturopathic preventive approach as illustrated earlier:

'People in the country are proud people... they don't like asking for your help... but they will go to the health food store and buy something that they can use themselves.' (EM)

Another informant explained how in her experience rural patients' need to feel in control and be central to decision-making in their healthcare, and how this influences her approach to providing naturopathic care:

'[Country people] are very strong... they do prefer their own counsel... you can't push them very much.... If they feel as though they are doing this themselves... then you have their compliance.... If you keep telling them what to do they don't really want to know. So I give them reading matter... handout sheets... they have a chance to go and think about it... seems to be a need to be in self-control.' (PE)

## Discussion

As this exploratory qualitative study highlights, rural naturopaths perceive and experience a range of affinities between their practices and the needs and qualities of rural patients and populations. It is interesting to note the participants' perceptions of the broad similarities between rural population's attitudes to health and the nature and practice of naturopathy.. Previous studies suggest that the combination of a number of push and pull factors may help explain higher CAM use in rural as compared to urban areas. These include differential access to care and health services, cultural norms and traditions of trust in CAM approaches to healthcare, lower income and health insurance in remote regions, high availability of CAM practitioners/practices in rural areas and differences in patient-practitioner interaction [16,17,28,30,35,48-53]. However, it is suggested that high use of CAM in rural areas may not be due to conventional care access issues alone as even in rural communities well-serviced by conventional services high CAM use is prevalent [17]. Cultural traits that have been associated with driving CAM use in rural populations include positive community connections (facilitating the flow of anecdotal information), increased levels of spirituality and attention to nutrition in rural communities [7]. Exploring the underlying drivers for cultural affinity for CAM services in rural areas can help develop policies and programs - both conventional and complementary - that are more culturally appropriate for rural communities. Examining the issue from a practitioner's point of view, this study confirms the importance of some of these push and pull factors in accounting for the rural patients' decision-making and health-seeking behaviour regarding CAM.

As an exploratory study, this research draws upon a self-selected sample of naturopaths in one region of rural Australia. The use of a self-selected sample may limit the generalisability of the respondents' observation on the respective practice, particularly when considering the variance created by the unregulated nature of the naturopathic profession in Australia. More research, both qualitative and quantitative, is needed to corroborate the findings of this study. Future studies should investigate the relationship and interface between naturopaths, patients and conventional providers in rural Australia [18]. Future research could also examine further the type of work naturopaths are undertaking in areas of restricted availability of conventional services as compared to those area well served by conventional services. In particular, it is important to examine the role of other primary care providers in the decision-making process surrounding rural naturopathic medicine use or non-use: how do they perceive naturopathic medicine and the use of naturopathic medicine by their patients? To what extent are these primary care providers informed about or enquire with their patients regarding the use of such medicine? And in what ways are they a support or a barrier to rural naturopathic medicine and broader CAM use in their practice community? Introducing data source triangulation through interviewing patients would also improve the reliability of these studies, move beyond this limitation of our study and help further understand the role of naturopathic practice in rural settings.

Similarly, there are a number of key questions regarding naturopathic medicine use and users in rural health care that remain to be investigated: How do rural naturopathic medicine users become introduced to such treatment options? And how do they make decisions regarding naturopathic medicine and a broader range of CAM treatments or providers and what are the characteristics of their pathways to care? Further exploration of these issues requires mixed-method designs utilising both national survey data and in-depth qualitative study of the experiences and perceptions of rural CAM users.

Further research is particularly important given the fact that naturopaths perceptions of their patients may not always compare with the normative data. For example, perceived affinity of rural population for preventive measures does seem to correlate with rural patients self-perceived notions of independence, stoicism, self-reliance and resilience [53-55] it does not seem consistent with epidemiological data relating to the higher prevalence of a number of preventive risk factors in rural areas [56]. These accounts of the naturopaths reveal the complexities of health-seeking behaviours in rural areas. Although rural patients have a relatively independent outlook and a natural affinity to prevention and self-management, these attitudes and affinities are not without their own ambiguities and they may in time act as a barrier deterring people from seeking professional (conventional or alternative) help. The stoical approach to health or life of the rural population, plus the shortage of healthcare service in remote regions, are important factors that may contribute to the higher prevalence of risk factors in rural areas relative to metropolitan areas.

Similarly, whilst the naturopaths in this study contrasted their practice and local and cultural knowledge with that of conventional medical practitioners, there are also many long-term conventional medical professionals practising in rural areas who would identify themselves as grounded by the needs of their practice and wider community and as developing good rapport and therapeutic relationships with their patients [57,58]. Although the patient-centred focus of CAM therapies has long been acknowledged as a 'pull' factor to CAM services in rural areas [2,10,48], or dissatisfaction from conventional medicine a 'push factor' for CAM consumption [51,52,59-61], it needs to be acknowledged that there are limitations to focusing on these as CAM-specific issues.

In addition to being Australia's largest CAM profession and largest unregistered health force, naturopaths are one of the fastest growing health professions [62] and they are well represented across many rural regions of Australia. Given the important and increasing role that naturopaths are playing in the Australian healthcare sector, there is surprisingly little research focused upon this practitioner group.

Naturopathic practice, defined by therapeutic philosophy rather than specific therapeutic tools [63], merits further investigation due to its increasing scope of practice and its potential impact may have on patients that increasingly rely on CAM therapists as primary care practitioners  [64]. The largely unregulated and undocumented nature of this significant component of healthcare delivery also challenges our ability to explore the potential impact of this sizable workforce on rural health outcomes.

The primary care role that naturopaths purport to play in these rural communities has implications for public health policy. Given the apparent community affinity for their services, naturopaths may be a potential resource for healthcare delivery in rural areas, particularly those underserved by conventional services. However, regulatory arrangements for naturopaths in Australia ensuring minimum standards of training and practice, as well as introducing practitioner accountability, may need to be considered if this potential role is to be explored further. Interestingly, all but one participant made comments supportive of naturopathic regulation in Australia, with the remaining respondent uncommitted.

## Conclusions

Further investigation of significant practice and policy issues is critical to understanding both the current and potential role of naturopathic medicine and its use in rural health care. Naturopathic medicine and CAM more generally, are becoming an increasingly mainstream health care option for many across Australia. Given the high levels of CAM use identified in rural areas both in Australia and overseas, it is imperative that naturopathy and the CAM sector more broadly become a core focus of rural health research.

## Competing interests

The authors declare that they have no competing interests.

## Authors' contributions

JW was involved with conception and design of the study, collected the data, analysing and interpreting the data and drafting and revising the manuscript; JA was involved with conception and design of the study, analysing and interpreting the data and drafting and revising the manuscript; C-WL was involved in analysing and interpreting the data and drafting and revising the manuscript. All authors read and approved the final manuscript.

## Pre-publication history

The pre-publication history for this paper can be accessed here:

http://www.biomedcentral.com/1472-6963/10/185/prepub

## Supplementary Material

Additional file 1 **List of interview questions**. A word document containing the list of interview questions in table format. Click here for file
