# Supplementary material for: A qualitative study of naturopathy in rural practice: A focus upon naturopaths' experiences and perceptions of rural patients and demands for their services
Source: BMC Health Serv Res. 2010 Jun 28;10:185. doi: 10.1186/1472-6963-10-185 (PMC2908615; doi:10.1186/1472-6963-10-185)
Supplement: Additional file 1 — List of interview questions. A word document containing the list of interview questions in table format. [file 1472-6963-10-185-S1.DOC]

**Additional File 1**

Additional File 1: List of interview questions

| 1. Please describe yourself and your role and responsibilities as a complementary medicine practitioner in this area. 2. What unique qualities related to the practice of complementary medicine exist in rural areas as opposed to other settings? 3. Do you think rurality affects complementary medicine use or demand for your services? If so how? 4. Is there something about the rural patient that affects their attitudes towards complementary medicine and your services? 5. Studies seem to suggest that rural populations use complementary therapies more than their urban counterparts. Does this surprise you and why, why not? 6. Have you experienced any barriers practicing complementary medicine in a rural setting? 7. What do you think needs to be done to overcome these barriers (if any)? 8. Describe your relationships with other conventional and complementary healthcare practitioners in the area. Does being a rural setting affect these relationships in any way? 9. What factors do you think are important to the successful practice and integration of complementary medicines in rural settings? 10. Are there any other factors that have shaped your experience as a complementary therapist in a rural area? |
| --- |
